# Evaluation of adverse drug reaction formatting in drug information mobile phone applications

**DOI:** 10.5195/jmla.2022.1251

**Published:** 2022-01-01

**Authors:** Sean M. McConachie, Dena Berri, Jewel Konja, Christopher A. Giuliano

**Affiliations:** 1 et6398@wayne.edu, Eugene Applebaum College of Pharmacy and Health Sciences, Wayne State University and Beaumont Hospital, Dearborn, Detroit, MI; 2 dena.berri@wayne.edu, PharmD Candidate, Eugene Applebaum College of Pharmacy and Health Sciences, Wayne State University, Detroit, MI; 3 iewel.konia@wayne.edu, PharmD Candidate, Eugene Applebaum College of Pharmacy and Health Sciences, Wayne State University, Detroit, MI; 4 ek2397@wayne.edu, Eugene Applebaum College of Pharmacy and Health Sciences, Wayne State University and Ascension St. John Hospital, Detroit, MI

**Keywords:** adverse drug reaction, clinical pharmacy information systems, drug information service

## Abstract

**Objective:**

To evaluate the differences in presentation (formatting) of adverse drug reaction (ADR) information within drug monographs in commonly used drug information (DI) mobile device applications.

**Methods:**

A cross-sectional analysis of ADR formatting of twenty commonly prescribed oral medications within seven DI mobile applications was performed. Databases were assessed for ADR information, including presence of placebo comparisons, severity of ADR, onset of ADR, formatting of ADRs in percentile (quantitative) format or qualitative format, whether references were used to cite information, and whether ADRs are grouped by organ system. Data was collected by two study investigators and discrepancies were resolved via consensus.

**Results:**

The seven DI mobile applications varied significantly on every analyzed ADR variable with the exception of ADR onset, which was absent in all databases. Significant differences were found for variables known to impact clinical judgment such as placebo comparisons and qualitative versus quantitative formatting. Placebo comparisons were most common among monographs in Lexicomp (30%) but were absent among monographs within other applications. Quantitative information was commonly used in most databases but was absent in Epocrates. Qualitative formatting was present in all Epocrates and Micromedex applications but absent in the majority of other applications. Substantial variations were also found in severity and grouping information.

**Conclusion:**

Substantial variation in ADR formatting exists among the most common DI mobile applications. These differences may translate into alternative interpretations of medical information and thus impact clinical judgment. Health care librarians and clinicians should consider ADR formatting when choosing between DI applications.

## INTRODUCTION

Adverse drug reactions (ADRs), which are defined as noxious and unintended responses to medications at normal doses, are common in clinical practice and contribute substantially to patient morbidity and mortality [1,2]. Observational studies suggest that ADRs occur in almost 17% of hospitalized inpatients, yet many ADRs are deemed to be potentially preventable by health care professionals [3–6]. The detection and prevention of ADRs remains difficult. It requires clinicians to make a causal assessment between a potentially offending medication and an adverse outcome, and the relationship is often muddied by the presence of numerous confounding variables [7]. Unlike other forms of adverse drug events, which may be mitigated with the use of drug information (DI) tools such as interaction checkers, alerts for prescribers, or other forms of pharmacovigilance, ADR detection remains difficult as it often requires more clinical judgment on the part of the health care provider [8]. However, the application of clinical judgment may also be troublesome as it is prone to individual subjectivity as well as a number of cognitive biases [8,9]. Clinical scoring tools such as the World Health Organization-Uppsala Monitoring Centre (WHO-UMC) and Naranjo scores have been developed to standardize the process of causal assessment for ADRs, but discordances often exist between the different scoring tools, and disagreement is common among clinical experts who assess potential ADRs [7,10,11]. With increasing numbers of medications being approved every year, the potential for new and significant ADRs continues to grow, as does the need for appropriate DI for clinicians who seek to prevent ADRs from occurring. For this reason, DI databases, which contain searchable information from medication package inserts, must be optimized for clinical use.

Health care providers are relying increasingly on DI databases in the form of applications on mobile devices to assist with clinical decision-making as smartphone adoption becomes more ubiquitous [12–14]. These databases are considered useful by many as they provide DI, including ADR information, at the point of care [15,16]. However, the presentation of ADR information in DI databases may predispose users to cognitive biases that can subsequently influence clinical judgment in ADR assessments. For example, it is widely known that framing effects, which are defined as differential interpretations of logically equivalent information, impact clinical judgment of both patients and health care professionals [17,18]. Presenting information in positive terms (survival) instead of negative terms (death), or as relative risks as opposed to absolute risks, can impact how medication risks and benefits are perceived [19,20]. Presenting comparative placebo information in combination with medication information also has been shown to influence patient perception of medication safety and effectiveness [21]. Other formatting differences, such as the use of qualitative versus quantitative formatting, can also influence comprehension of medication risk depending on an individual's numeracy [22,23]. A recent analysis found numerous differences in ADR formatting between DI applications that are available on desktop computers with regard to many criteria that are used by clinicians in causality assessments, including information regarding onset of ADR, severity of ADR, the presence of placebo comparison data, and frequency formatting [24]. However, there is no research available on the formatting of ADRs in DI mobile applications, which are used by a growing number of clinicians. In order for librarians and clinicians to ensure availability of appropriate DI applications in the setting of limited resources, educate future health care professionals, and identify the best available DI for the situation at hand, it is important that references optimize ADR formatting for unbiased clinical interpretation. Therefore, the objective of this study is to evaluate the differences in formatting of ADR information within drug monographs in commonly used DI databases in mobile device applications.

## METHODS

This was a cross-sectional study to evaluate the framing of ADR information within drug monographs from commonly used DI mobile applications. Seven electronic DI databases were selected for analysis, which included Micromedex, Epocrates, Lexicomp, Clinical Pharmacology, Medscape, Drugs.com, and Pocket Pharmacist. These databases were chosen due to research suggesting their high prevalence in clinical practice, functionality as DI databases, and availability as mobile applications [25]. Database analysis was performed during the period of September 7 to December 31, 2020.

The twenty most highly prescribed medications in the United States, as listed by ClinCalc.com (as of December 1, 2020), were chosen to compose the sample drug monograph population, as was done in a previous study [24]. A list of the DI mobile applications and assessed medications is available in Table 1. Each ADR section within the applications' drug monographs was evaluated for the following criteria in the order presented: quantitative (number-based) versus qualitative (word-based) formatting of ADR frequency, presence or absence of comparative placebo ADR frequencies, severity assessment for ADRs, onset information for ADRs, grouping of ADRs by organ system, and the presence or absence of references for ADR information. Definitions for each criteria are provided in Appendix 1. These criteria were chosen because each is either important in risk framing (quantitative/qualitative format, presence of placebo information), integral in making an ADR causality assessment (timing and severity of ADR information, presence of placebo information), or potentially important for ease of use of ADR information as assessed by study investigators (grouping of ADRs by organ system).

**Table 1:** Evaluated medications and drug information databases

Top 20 oral medications in the US	Drug information databases
Levothyroxine	Micromedex
Lisinopril	Epocrates
Atorvastatin	Lexicomp
Metformin	Clinical Pharmacology
Amlodipine	Drugs.com
Metoprolol tartrate	Pocket Pharmacist
Omeprazole	Medscape
Simvastatin	
Losartan	
Albuterol	
Gabapentin	
Hydrochlorothiazide	
Acetaminophen-hydrocodone	
Sertraline	
Furosemide	
Fluticasone	
Acetaminophen	
Amoxicillin	
Alprazolam	
Atenolol	

Additionally, these criteria correspond to the Food and Drug Administration's guidance for manufacturer labeling of adverse reactions in package inserts [26]. Following assessment of all medication monographs within each database, an average across the twenty medications for each category was conducted for the database as a whole to obtain mean values for each ADR variable within a given application.

Two investigators (DB and JK) assessed the variables for each of the twenty medication monographs across the DI databases. Agreement between investigators was evaluated for all datapoints. Discrepancies were resolved via consensus by reviewing the datapoints and monograph in question and receiving input from a third investigator (SM). The chi-square test was used to evaluate for between-group differences in categorical variables.

## RESULTS

Every medication (n=20) contained a representative monograph in each of the DI mobile applications, allowing complete analysis across all variables and drugs (Table 2). During collection, there were 16 discrepancies across the 980 collected datapoints (interrater agreement=98.4%). All discrepancies (100%) were resolved via consensus and reassessment of the datapoint in question. Statistically significant differences were detected between DI mobile applications for each of the analyzed ADR variables (*p*< 0.01), with the exception of ADR onset information (analysis unable to be conducted as the variable was absent in every DI application).

**Table 2:** Analysis of drug information formatting in mobile applications

Database^*^ ADR variable	Micromedex	Epocrates	Lexicomp	Clinical Pharmacology	Drugs.com	Pocket Pharmacist	Medscape	Significance
**Placebo comparison**	0%	0%	30%	0%	0%	0%	0%	X^2^ (6) = 37.6; p<0.01
**Severity assessment**	100%	100%	0%	0%	0%	0%	0%	X^2^ (6) = 140.0; p<0.01
**Onset of ADR**	0%	0%	0%	0%	0%	0%	0%	*
**Quantitative frequency**	85%	0%	80%	95%	85%	90%	70%	X^2^ (6) = 64.1; p<0.01
**Qualitative frequency**	100%	100%	0%	95%	90%	0%	5%	X^2^ (6) = 125.0; p<0.01
**References provided**	0%	0%	100%	0%	100%	100%	0%	X^2^ (6) = 140.0; p<0.01
**Organ system grouping**	100%	0%	100%	0%	100%	0%	15%	X^2^ (6) = 129.7; p<0.01

* All variables are presented as mean percentages. Each formatting variable was assessed in twenty medications in every analyzed mobile application. Percentages refer to the number of monographs that contained the particular formatting variable divided by the total number of analyzed monographs (n = 20) in each database.

Inclusion of placebo information was rare in DI mobile applications. Only Lexicomp monographs contained placebo information, and these comparisons were present in the minority of monographs (30%). Every other application did not contain placebo information in the ADR section of any of the analyzed monographs. Likewise, ADR severity information was rare across platforms. Epocrates and Micromedex included notation of ADR severity in all analyzed monographs (100%); however, severity assessments were completely absent in the remaining databases (0%). As mentioned above, none of the analyzed databases included information on ADR onset.

Frequency formatting of ADR information in DI applications varied widely (*p*<0.01). Quantitative frequency was present in the majority of monographs in Clinical Pharmacology, Pocket Pharmacist, Micromedex, Drugs.com, Lexicomp, and Medscape, although there were numeric differences between platforms. Only in Epocrates was quantitative formatting completely absent. On the other hand, qualitative frequency was used in all analyzed monographs within Epocrates and Micromedex and was also present in high proportions in Clinical Pharmacology and Drugs.com. It is worth noting that several databases (Micromedex, Clinical Pharmacology and Drugs.com) format references using a combination of qualitative and quantitative formats.

References for ADR information were provided in all monographs (100%) within Lexicomp, Drugs.com, and Pocket Pharmacist but were absent (0%) in the remaining databases. Finally, the analyzed databases varied widely with regard to whether or not ADRs were grouped based on organ system (*p*<0.01). Micromedex, Lexicomp, and Drugs.com used organ system grouping in all the analyzed monographs (100%), whereas organ system grouping was present only in a minority (15%) of Medscape monographs and entirely absent from Epocrates, Clinical Pharmacology, and Pocket Pharmacist.

## DISCUSSION

This study demonstrates that the presentation of ADR information in DI mobile applications is inconsistent for the most commonly prescribed medications in the United States. This is problematic as differences in information formatting are known to impact clinical judgment and decision-making [17,18,20]. Recent surveys suggest that up to 74% of pharmacy students and over 60% of medical residents use health care-related mobile applications on a consistent basis [25]. These trends are likely to increase as smartphone usage becomes more widespread. The prevailing differences in formatting uncovered by this research, in addition to prior research that has identified that formatting of ADR sections is an independent modifier of clinical judgment in both pharmacists and pharmacy students, suggest that health care providers may come to different decisions in clinical situations in which potential ADRs are present [27]. This creates a problem for health librarians, health care providers, and teachers of evidence-based medicine who are charged with identifying unbiased sources of DI for clinicians in practice.

Similar to a previous study of DI desktop applications, we identified variability across DI mobile applications in a variety of formatting parameters that are known to influence clinical decision-making [24]. Mobile applications varied substantially with regard to the presence of comparative placebo information, ADR severity information, referencing, organization by organ system, and both quantitative and qualitative formatting. Many of these components are integral to ADR evaluation. For example, assessments of potential ADRs in practice require clinicians to assess the probability that an adverse outcome was the result of a drug as opposed to concomitant disease states or other factors [10,11,28]. Comparative placebo information is an important factor in distinguishing between a drug-induced or disease state-induced event, and ADR detection may be more difficult when comparative information is absent. In one previous study, patients were randomized to receive efficacy information about a fictious medication with or without accompanying placebo information [21]. Patients who received placebo information tended to perceive the drug as less effective than those who did not receive the information. The incorporation of placebo information into other contexts of medication outcome presentation has already been recommended by physicians [21].

The formatting of ADR frequency was also different between databases. Although empirical research in clinicians is lacking, health communication researchers generally recommend quantitative rather than qualitative formatting of frequencies, as quantitative information gives a more detailed assessment of risk [22,23,29]. This would likely be beneficial from a clinical perspective, especially with point-of-care tools such as mobile DI databases. For example, evaluating whether a reaction represents a potential ADR could be made easier with quantitative, rather than qualitative, frequency information. Furthermore, incorporation of quantitative ADR information with placebo or active comparator information from clinical trials allows clinicians to compare absolute risks, leading to more informed clinical decision-making. For example, clinical decision-making processes could be significantly altered if a user decided to use Epocrates (which labels ADRs that occur at a frequency of greater than 3% under the ambiguous label “common”) or Micromedex (which uses both quantitative [percentages] as well as qualitative [“common”] formatting for ADR frequencies). Every other DI mobile application utilized quantitative formatting in the majority of analyzed drug monographs.

The broader clinical consequences of the variability of ADR formatting within DI databases, across both mobile and desktop applications, is still unknown. Numerous previous analyses have identified significant differences regarding both the content and usability of DI databases across a variety of factors including scope, completeness, and ease of use [30]. Yet, there has been relatively little attention paid to information formatting. However, the inconsistencies across databases beg the question of whether and how DI databases should be regulated. For the time being, the Food and Drug Administration regulates only medical applications that are interpreted to be medical devices. That is, applications that are designed “for use in the diagnosis or the cure, mitigation, treatment, or prevention of disease, or to affect the structure or any function of the body of man” [31]. Other mobile applications, including those pertinent to DI questions, are not subject to the same scrutiny. In the absence of regulatory oversight, studies which seek to provide consensus across health care disciplines, health communication, and clinical psychology could be helpful in optimizing DI databases for use in clinical practice. Without such studies, DI database companies can continue to structure their products according to their own opinions and whims. Until such studies are conducted, health librarians and clinical practitioners should be wary of the limitations inherent in DI databases on their mobile devices and strive to reference more than one database each time DI questions arise in practice.

This study, like previous research that has suggested variability in clinically pertinent outcomes between DI databases, suggests that future research aimed at DI database optimization is critical given current gaps in knowledge. For example, this study was unable to discern optimal formatting of ADRs for clinical practice. Currently, the best method of information formatting to implement in DI databases or mobile applications is unknown. Research that suggests framing effects impact clinical judgment are abundant, but studies that analyze which method of risk framing optimizes clinical judgment are lacking. Some aspects of ADR formatting, such as severity assessments, organ system grouping, and the need for references, will likely remain open to interpretation and would benefit from analyses of user preference, as described above.

This study has a number of limitations. First of all, this project, similar to the previous analysis of DI databases, was limited to an assessment of twenty medications [24]. However, by evaluating the most widely prescribed medications in the United States, including drugs that were approved over disparate periods of time (and thus under varying degrees of regulatory approval by the Food and Drug Administration), we believe the information drawn from the monographs within the analyzed mobile applications is likely a good representation of ADR formatting more generally [32]. Additionally, the study only evaluated eight mobile DI applications. There is currently no way to reliably estimate how many DI applications are available to users, due to the lack of regulations on applications of this type [25]. However, we studied the applications that have been cited as the most prevalent in clinical practice. We believe the variability demonstrated in this study likely extends to the numerous other DI applications in existence. Despite these limitations, this study does provide a characterization of the wide variability of framing effects in commonly used mobile applications, which can provide a starting point for future research on the optimization of DI applications.

In conclusion, formatting of ADR information within commonly used DI mobile applications is inconsistent. The differential presentation of ADR information may impact clinical judgment and decision-making. Health care librarians and clinicians should consider ADR formatting when choosing between DI applications.

## Data Availability

Data associated with this article are available in the Open Science Framework athttps://osf.io/pjcqy/.

## SUPPLEMENTAL FILES

**Appendix 1.** Adverse drug reaction criteria definitions
