# Supplementary material for: Evaluation of adverse drug reaction formatting in drug information mobile phone applications
Source: J Med Libr Assoc. 2022 Jan 1;110(1):81–6. doi: 10.5195/jmla.2022.1251 (PMC8830402; doi:10.5195/jmla.2022.1251)
Supplement: Supplementary file 1 — Appendix 1. Adverse drug reaction criteria definitions [file jmla-110-1-81-s01.docx]

**Appendix 1: Adverse drug reaction criteria definitions**

| **ADR Criterion** | **Definition** | **Example Use** |
| --- | --- | --- |
| **Quantitative frequency** | Frequencies provided in numeric format | Gastrointestinal upset (14%) |
| **Qualitative frequency** | Frequencies provided in word-based format | Common ADRs  Gastrointestinal upset |
| **Comparative placebo frequency** | Frequencies provided for both patients who received medication and those who received placebo | Gastrointestinal upset (14% in medication group; 7% in placebo group) |
| **Severity information** | Monograph stratifies ADRs by level of potential harm | Non-severe reactions:  Gastrointestinal upset |
| **Onset information** | Monograph details when ADR presents following initiation of medication | Gastrointestinal upset (most common within 6 months of initiation) |
| **Grouping by organ system** | Monograph separates ADR information based on affected organ system | Gastrointestinal system:  Gastrointestinal upset, diarrhea |
| **References** | Monograph cites ADR information using references to specific sources | Gastrointestinal upset^1^  References:  1. Medication package insert (2020). |
